# Design of an Epitope-Based Vaccine Against MERS-CoV

**DOI:** 10.3390/medicina60101632

**Published:** 2024-10-06

**Authors:** Taghreed N. Almanaa

**Affiliations:** Department of Botany and Microbiology, College of Science, King Saud University, Riyadh 11451, Saudi Arabia; talmanaa@ksu.edu.sa

**Keywords:** Middle East Respiratory Syndrome (MERS), epitope vaccine, molecular docking, molecular dynamic simulation

## Abstract

*Background and Objectives:* Middle East Respiratory Syndrome (MERS) is a viral respiratory illness caused by a coronavirus called Middle East respiratory syndrome. In the current study, immunoinformatics studies were applied to design an epitope-based vaccine construct against Middle East Respiratory Syndrome. *Materials and Methods:* In this study, epitopes base vaccine construct was designed against MERS using immunoinformatics approach. *Results:* In this approach, the targeted proteins were screened, and probable antigenic, non-allergenic, and good water-soluble epitopes were selected for vaccine construction. In vaccine construction, the selected epitopes were joined by GPGPG linkers, and a linear multi-epitope vaccine was constructed. The vaccine construct underwent a physiochemical property analysis. The 3D structure of the vaccine construct was predicted and subjected to refinement. After the refinement, the 3D model was subjected to a molecular docking analysis, TLRs (TLR-3 and TLR-9) were selected as receptors for vaccine construct, and the molecular docking analysis study determined that the vaccine construct has binding ability with the targeted receptor. *Conclusions:* The docking analysis also unveils that the vaccine construct can properly activate immune system against the target virus however experimental validation is needed to confirm the in silico findings further.

## 1. Introduction

Middle East Respiratory Syndrome (MERS) is a viral respiratory illness caused by the Middle East respiratory syndrome coronavirus (MERS-CoV) [1]. The virus can be transmitted from camels to humans through direct physical contact, with limited human-to-human transmission also possible. Most diagnosed cases of MERS have resulted in severe respiratory disease, leading to high mortality and morbidity rates [2]. While the majority of MERS cases have been reported in countries within or near the Arabian Peninsula, some travel-related cases have been identified in countries outside of this region [3]. In some instances, MERS may be asymptomatic. However, symptoms typically begin within 1 to 2 weeks after infection, often around 5 days post-exposure, but they can manifest up to 14 days later [4]. Common symptoms of MERS include fever, chills, coughing, sore throat, runny nose, difficulty breathing, and muscle aches.

Immunoprophylaxis against viral illnesses involves the use of vaccines or antibody-containing preparations to provide immunologic protection to susceptible individuals against specific diseases. Immunization can either be active or passive [5]. Active immunity is achieved by stimulating the body’s immune response. Reverse vaccinology is a novel approach to vaccine design that leverages the rapidly advancing ability to sequence entire genomes of microorganisms and apply bioinformatics analyses to the data [5]. Predictive modeling is used to identify new pathogen targets that are ideally conserved and elicit protective responses. These candidate targets are then expressed and screened using human serum from individuals with effective immunity and evaluated in murine models [6]. This process can lead to the development of optimal vaccines, especially against fatal viruses [7]. The use of vaccines has led to a significant improvement in global health, saving numerous lives, reducing treatment costs, and enhancing the quality of life for both humans and animals [8]. Traditional vaccines were developed empirically, often with little to no understanding of how they modulate the immune system [9]. Despite advancements in vaccine design, there are still immune-related concerns, particularly in specific vulnerable populations and in cases of emerging or re-emerging infectious diseases [10].

## 2. Research Methods

**Figure 1:**
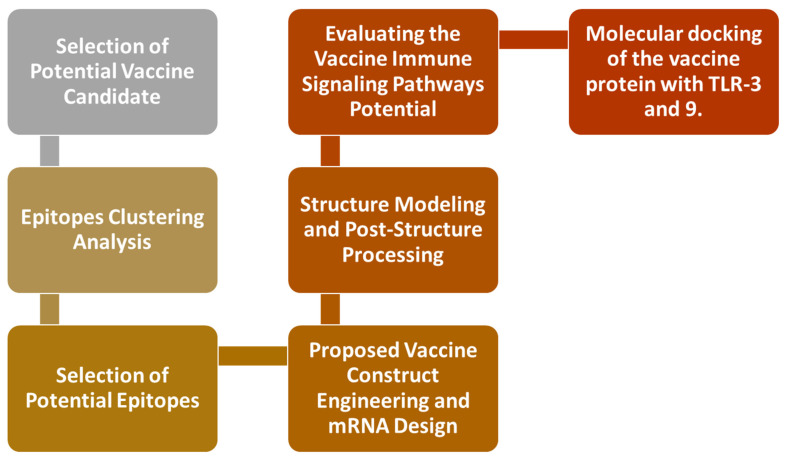
A schematic diagram illustrating the construction and processing of a multi-epitope vaccine. This study begins with the selection of a MERS-specific spike protein from a biological database. Epitopes were then predicted from the query protein and prioritized using various machine learning classification models. The filtered epitopes were linked together to form a complete three-dimensional structure, which was subsequently subjected to immune simulations to predict the immune response against the vaccine construct. The resulting vaccine candidate, which demonstrated potential immune stimulation and structural stability, was further evaluated for performance through docking studies with known immune receptors.

The research flow is given in Figure 1.

The MERS spike protein (accession id: K9N5Q8 · SPIKE_MERS1) was selected from the UniProt database and retrieved for an immunoinformatic analysis [11]. To find all the reported epitope datasets of the protein, the IEDB Epitope Source platform [12] was utilized, where the spike protein served as an antigen, while MERS was considered as an organism. In the epitope section, “Any Epitope” was opted, which includes linear, discontinuous, and non-peptide epitopes. In the Assay tab, the T cell, B cell, and MHC ligand options were selected [13]. In the “MHC Restriction” window, any option was opted for, including Class I, Class II, and non-classical. In the case of “Host Tab”, the Human host was selected. By doing so, 3474 epitopes were identified. After thoughtful investigation, the duplicate epitopes were discarded, and only single and non-redundant epitopes were selected for additional analysis. The database can be accessed at https://www.uniprot.org/uniprotkb/K9N5Q8/entry (accessed on 1 July 2024) spike glycoprotein OS = Middle East respiratory syndrome-related coronavirus; OX = 1335626; GN = S; PE = 3; SV = 1.

### 2.1. Epitopes Clustering Analysis

The collected set of epitopes was then subjected to an epitope cluster analysis using IEDB Epitope Cluster Tool v2.0 [14]. This tool identifies a group of epitopes while considering sequence identity. The identity cut-off was set to 70%. The maximum and minimum lengths of the epitopes were not required. The clustering algorithm used involved breaking the connected clusters to obtain a clear consensus sequence.

### 2.2. Selection of Potential Epitopes

The representative and consensus epitopes from the epitope clusters were then investigated for several different checks such as antigenicity, allergenicity, toxicity, homology against human proteome, IFN-gamma production, and binding affinity for DRB*0101 alleles. The DRB1*0101 allele is one of the alleles of the HLA-DRB1 gene, part of the Human Leukocyte Antigen (HLA) system which encodes proteins involved in presenting antigens (peptides derived from pathogens or self-proteins) to CD4+ T cells [14]. The antigenicity of the opted epitopes was assessed using VaxiJen v2.0 [15]. The target organism selected was a virus, and the antigenicity cut-off was allowed to be set as default (>0.5) [8,16,17,18]. VaxiJen is an online server designed to predict protective antigens and subunit vaccines. The allergenicity of the antigenic epitopes was investigated using AllerTOP v2.0 [19]. The server uses auto cross-covariance transformation of the given peptide sequence into uniform equal-length vectors. The epitopes are classified either as allergen or non-allergen. Next, the selected epitopes were analyzed for toxicity, which was carried out using the ToxinPred online server [20]. The non-toxic epitopes were selected for further analyses. For comparative homology analysis, the shortlisted epitopes were subjected to homology analysis against the human proteome with a sequence identity value of ≤30% and a bit score of 100%. The selection of interferon-gamma (IFN-γ) epitopes is key in designing potent vaccine candidates as it helps provide crucial CD4+ T helper cell and CD8+ T cytotoxic cell immunity against the infectious pathogen. Finally, MHCphred analysis was performed to evaluate the epitopes’ ability to interact with the DRB*0101 allele [21,22]. This allele is predominant in the human population; thus, any epitope interacting with the allele can lead to the formation of robust and targeted protective immune responses.

### 2.3. Proposed Vaccine Construct Engineering and mRNA Design

The vaccine construct of the mRNA sequence was designed in the following order from the N to C terminus: m7GCap and UTR at the 5′ end, signal peptide, linker (EAAAK), RpfE adjuvant, Linker (GPGPG), CD4+helper T lymphocytes, linker (AYY), CD8+ cytotoxic T lymphocytes, MITD sequence, stop codon, and UTR-poly A tail at 3′ end [23].

### 2.4. Structure Modeling and Post-Structure Processing

The proposed vaccine sequence was then used for 3D structure construction using the 3Dpro online server in SCRATCH. The vaccine structure was further improved by modeling the structure loops, followed by refinements using the GalaxyLoop and GalaxyRefine servers, respectively [24]. To increase the structure strength, disulfide bonds were introduced into the structure using Design 2.0 [25]. The vaccine sequence was then reverse-translated to nucleotide so it could be cloned into an pET-28a(+) expression vector [26]. This was achieved using the SnapGene software.

### 2.5. Evaluating the Vaccine’s Immune Signaling Pathway Potential

The profiling of generated immune responses by the designed vaccine was carried out using C-ImmSim server simulations [27]. The tool is often best when used with other tools like Vaxim and SIMVACS because it works on an agent-based model where the vaccine sequence is considered as an antigen and employs the position-specific scoring matrix algorithm for predicting immunological responses. The immune reactions and interactions with the vaccine antigen were then modeled through machine learning algorithms. The immunological responses were simulated in three anatomical compartments such as tertiary lymph nodes, thymus, and bone marrow. During this simulation analysis, the number of simulation steps set was 1000, the random seed value was set to 12,345, and simulation volume value was 10 [28].

### 2.6. The Molecular Docking of the Vaccine Protein with TLR-3 and 9

Molecular docking analysis was carried out in order to analyze the binding ability of the vaccine with immune cell receptor; for the docking analysis, cluspro 2.p webserver was used, and population coverage analysis was carried out using the IEDB database (http://tools.iedb.org/population/) (accessed on 15 July 2024) [29].

## 3. Results and Discussion

The epitopes were retrieved from IEDB and subjected to an immunoinformatics filter as mentioned in Table 1, and only probable antigenic, non-allergenic, good water-soluble, and IFN-gamma inducers were selected for vaccine designing in order to activate the proper immune system against the target pathogen. These epitopes were selected using machine learning-based classification models trained on biological epitopes that were confirmed through lab experiments. The query genome of the mentioned virus was processed through the trained machine learning model incorporated in the IEDB database, which predicted the most significant epitopes from the genome. Furthermore, the epitopes were prioritized based on their chemical and biological properties, such as antigenicity, toxicity, allergenicity, and MHC binding, using various tools trained on confirmed biological amino acid sequences with known properties. This approach of selection was used by numerous research articles presenting supported results and conclusions [30,31,32].

### 3.1. Multi-Epitope Vaccine Construction and Refinement

**Figure 2:**
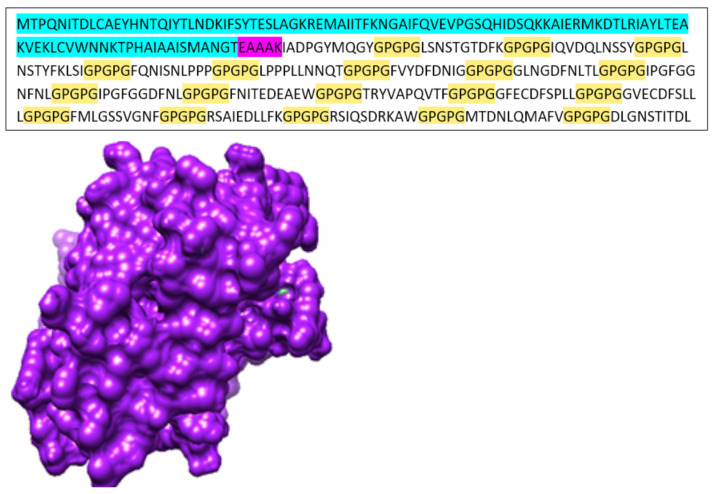
This figure presents the sequence of the vaccine construct, where the adjuvant is highlighted in blue, the linkers are in yellow, and epitopes are not highlighted. Furthermore, the 3D structure of vaccine was modeled. This model explains a highly packed, completely modeled structure vaccine construct with multiple grooves, predicting the effective binding of immune receptors.

The selected epitopes were linked by GPGPG linkers and further connected to an adjuvant, generating a linear sequence. In the vaccine construct, the epitopes were arranged such that the B cell epitopes appeared first, followed by the T cell epitopes. The obtained sequence was then used for structural modeling. The structure was refined by galaxyweb, and the server generated the top 5 models based on different parameters; the top 1 structure was selected for docking analysis. Overall, the refinement results are presented in Table 2, and the refined structure plus the exact sequence of the vaccine construct are presented in Figure 2. The GPGPG linkers are mostly used in constructing in silico vaccine constructs because they make the vaccine more rigid and highly exposed to immune receptors and they avoid self-complementarity between used epitopes [33]. This type of linker has been used by multiple authors and has shown to be effective, yielding promising results. Furthermore, the modeled vaccine construct was structurally refined to make it more relaxed and highly stable for further analysis. This approach is commonly used to prepare a vaccine for docking studies. Galaxy-web employs highly accepted force fields, where the structure is examined and relaxed under all possible conditions, particularly considering the bond angles between the amino acids [34,35,36].

### 3.2. Disulfide Engineering

**Figure 3:**
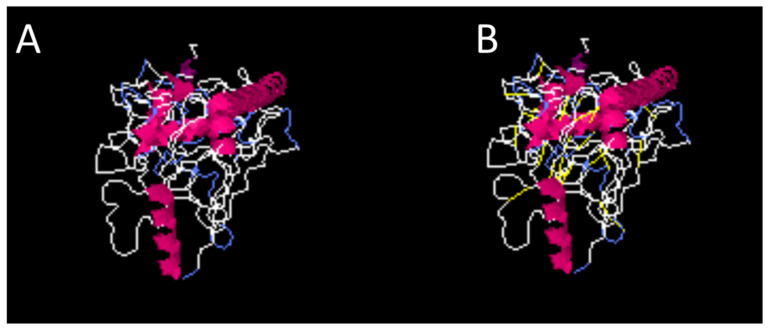
The wild-type (**A**) and mutated structure (**B**) of the vaccine construct. In the mutated vaccine structure, yellow represents di sulfide bonds. In both structures, white shows the loop, blue shows the sheet, and pink shows the ribbon secondary structure elements.

Disulfide engineering was carried out to stabilize the structure of the vaccine construct further; the amino acids’ positions and pairs, chi 3 values, and energy are presented in Table 3. Furthermore, Figure 3 presents the mutation and wild type of the vaccine construct. Disulfide by Design 2.0 is one of the most significant biotechnological webservers that is used in advanced research; the incorporation of disulfide bonds can improve the stability of the protein [37,38,39].

### 3.3. Molecular Docking Analysis

**Figure 4:**
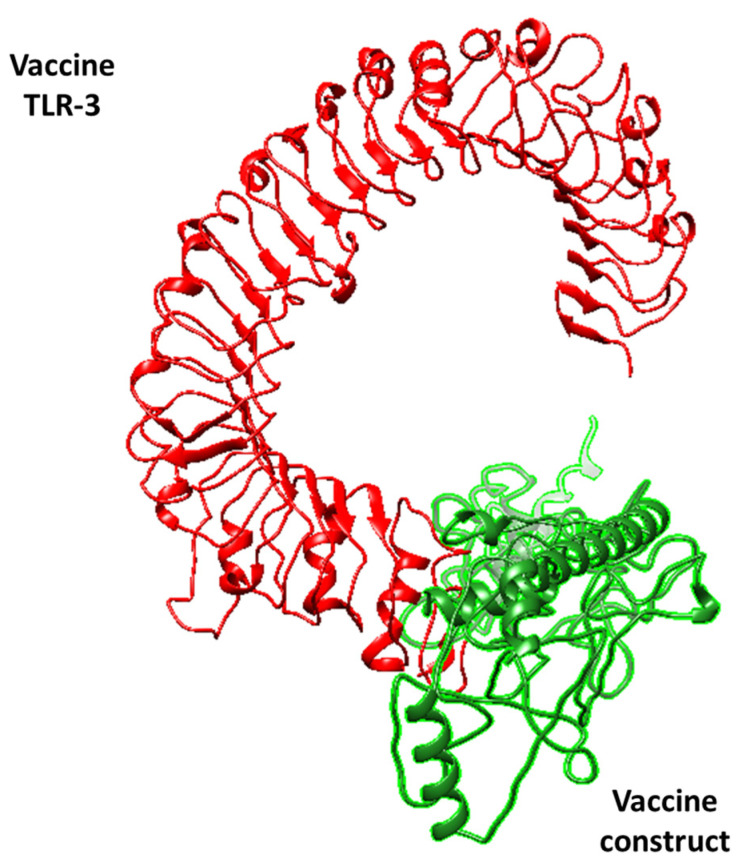
The Vaccine_TLR-3 complex. The red color represents the TLR-3 candidate, while green represents the vaccine. The figure shows that the vaccine is purely docked to the side domain of TLR-3, thus showing effective binding.

**Figure 5:**
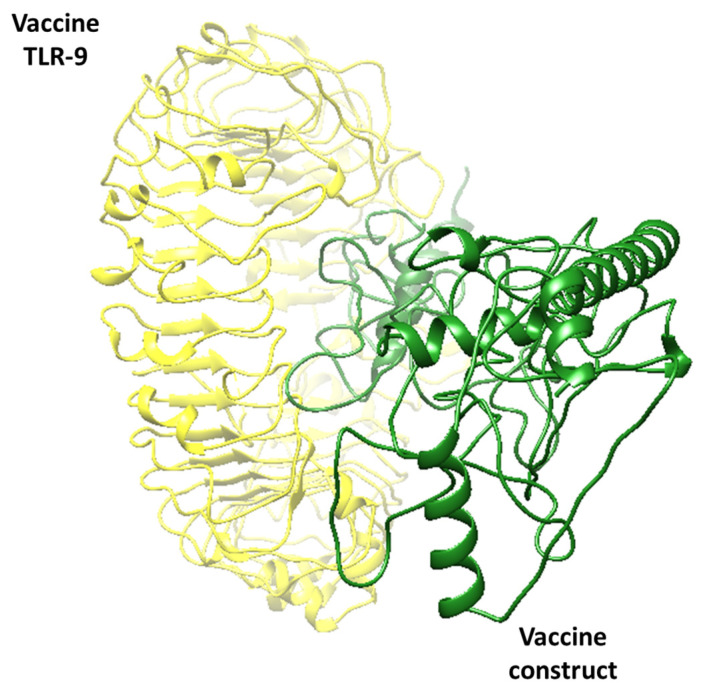
The Vaccine_TLR-9 complex, where the yellow color represents TLR-9, while the green color represents the vaccine candidate. The figure shows that the vaccine is purely docked to the middle region of TLR-9, thus showing effective binding.

As mentioned, an effective vaccine candidate should have the potential for significant binding efficiency with an immune receptor. This will enable the immune system to recognize it and trigger a robust and effective immune response against the vaccine. To ensure the effectiveness of our designed vaccine, docking studies were performed where the vaccine was virtually docked to the TL3 and TL9 proteins that are present on the surface of host macrophages. This was carried out on purpose because these immune receptors almost have the potential to bind to foreign substances, thus promoting a series of immune reactions [40]. These receptors are mostly used in vaccine designing studies where scientists check the efficacy of their proposed vaccine [41]. Upon docking, the results reveal that the vaccine and target receptors have proper binding ability, indicating that the vaccine construct can activate an immune response against the target pathogens. ClusPro 2.0 generated docked complexes, as shown in Table 4 and Table 5. Additionally, the docked conformations are illustrated in Figure 4 and Figure 5. The docking results suggest that the vaccine can effectively interact with the target receptor and trigger an immune response against the pathogen.

### 3.4. Host Immune Simulation

**Figure 6:**
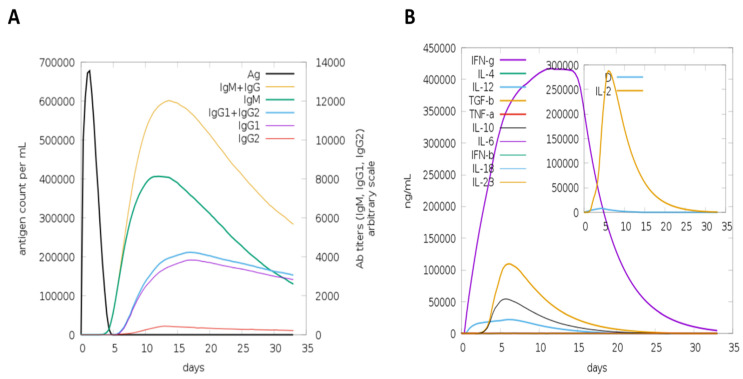
Antibody and cytokine levels toward multi-epitope vaccine construct. (**A**) shows that upon the introduction of the vaccine antigen (black curve), starting from day 5, the adaptive immune response was highly activated. Large amounts of IgM and IgG, along with their subtypes, were produced in response to the vaccine candidate, which is a typical reaction when a foreign pathogen enters the body. (**B**) The results show cytokine production against the vaccine candidate. Large amounts of IFN-gamma along with other cytokines are produced, which clearly represent the robust activation of the immune system against the vaccine construct.

**Figure 7:**
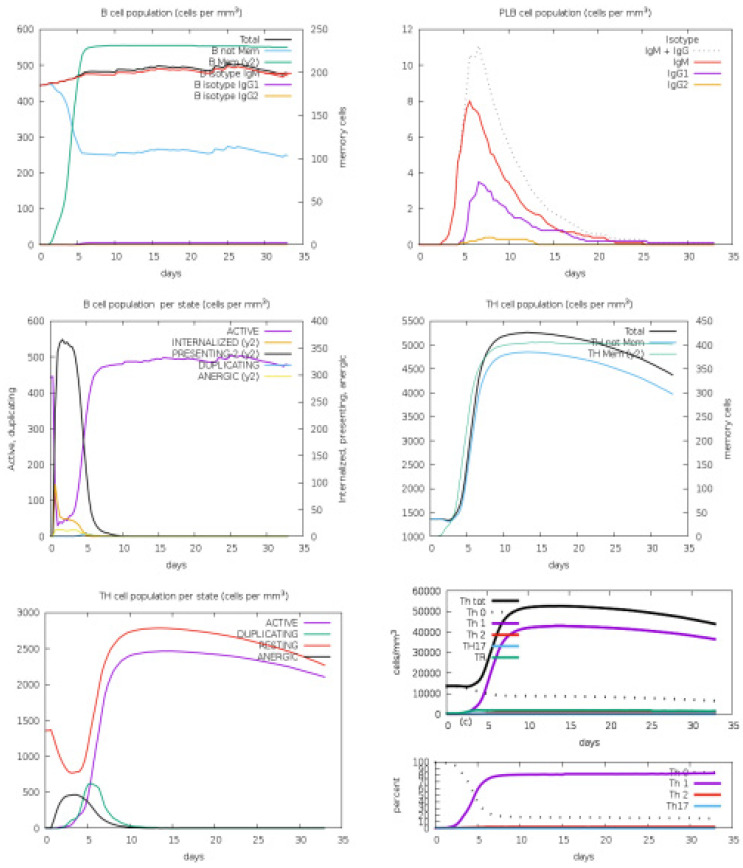
Immune simulation reports. Legend: Act = active, Intern = the internalized Ag, Pres II = presenting on MHC II, Dup = in the mitotic cycle, Anergic = anergic, Resting = not active.

**Figure 8:**
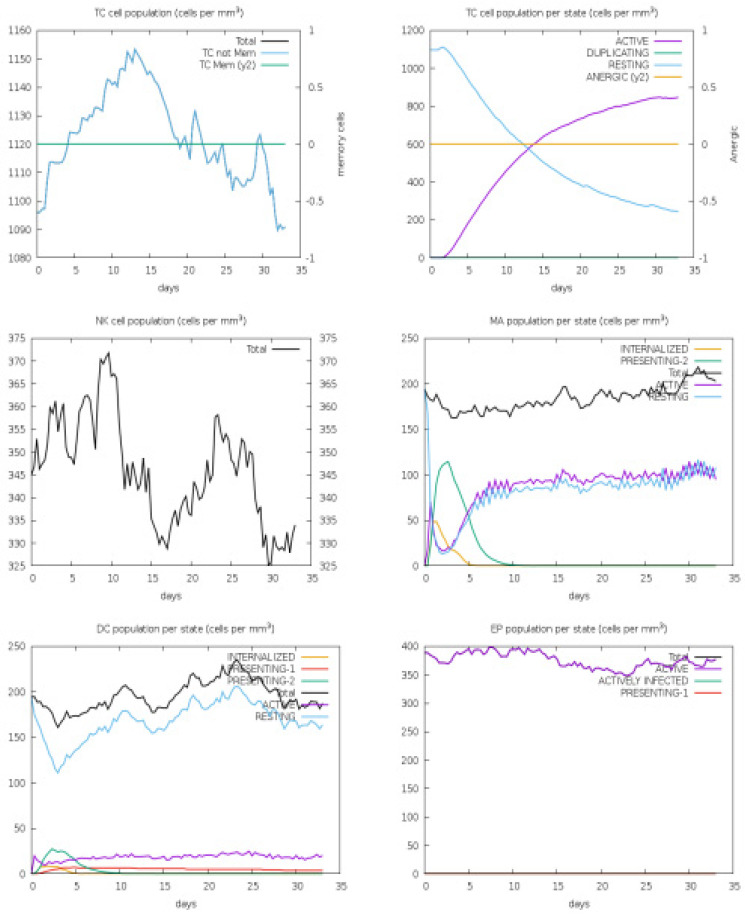
Immune simulation reports. Legend: Act = active, Intern = the internalized Ag, Pres II = presenting on MHC II, Dup = in the mitotic cycle, Anergic = anergic, Resting = not active.

To confirm the significance of the designed vaccine candidate, immune simulations were conducted to observe the extent of the immune response it provoked. This model is trained on antigen-based proteins and can predict the magnitude of immune responses triggered by the query antigens. In the human C-immune simulation analysis, various antibodies and cytokines were observed in response to the vaccine construct. IgM and IgG were reported in high concentrations, followed by IgM1 and IgG2. Among the cytokines, IFN-gamma was observed in the highest concentration, followed by IL-4, IL-12, TGF-b, TNF-a, IL-10, IL-6, IFN-b, IL-18, and IL-23, as represented by different colored peaks. Figure 6 present different antibodies and cytokines generated in response to the vaccine construct. Furthermore, the overall immune responses are depicted in Figure 7 and Figure 8.

## 4. Conclusions

Designing and developing vaccines is a complex process, but computational immunology techniques have the potential to significantly reduce the workload. Epitope-based vaccines are both feasible and effective in eliciting a protective immune response. In this study, we predicted B and T cell epitopes for target pathogens, and the identified minimal epitope sets may serve as promising vaccine candidates for future use. The population coverage analysis suggests that the proposed epitopes could be effective across a significant portion of the human population. Overall, the immunological analysis, along with structural and physicochemical characterizations, indicates that the vaccine candidate requires further in vitro and in vivo validation.

## Figures and Tables

**Table 1:** Epitopes and their immunoinformatics screening. The selected peptides in the table were screened through machine learning classification models which predict and select the most significant peptides that might have confirmed mentioned properties (for detail, see text). The mentioned epitopes have properties in an accepted range that could withstand the normal desired properties of a normal highly efficient epitope. The epitopes in the table are ranked based on antigenicity score in descending order. NA: not applicable.

Selected Peptide	Type	Start and End Amino Acid	Antigenicity	Allergenicity	Toxicity	Homology	IFN-Gamma Peptide
FVSKDVKFENLTNLPPPLLN	Consensus	101–119	1.1267	Probable Non-Allergen	No	No	No
LDAGLNGDFNLTLLQVP	Consensus	625–642	0.8180	Probable Non-Allergen	No	No	Yes
CDNYDDYDFEPQKI	Consensus	687–702	1.4164	Probable Non-Allergen	No	No	Yes
CPMMDLGNSTITDLGV	Consensus	135–151	1.2037	Probable Non-Allergen	No	No	Yes
CCDYEEYDLEPHK	Consensus	745–758	1.1076	Probable Allergen	NA	NA	NA
ITTLNTRYVAPQVTF	Consensus	514–528	1.0017	Probable Non-Allergen	No	No	Yes
LKNRCCDRYEEYD	Consensus	457–469	0.9942	Probable Allergen	NA	NA	NA
ISPAAISSNCYS	Consensus	320–333	0.9397	Probable Allergen	NA	NA	NA
LFSVNDFTCSQISPAAIASN	Consensus	945–965	0.8997	Probable Allergen	No	No	Yes
EEYDLEPHKIHVH	Consensus	638–650	0.8688	Probable Non-Allergen	No	No	Yes
FMHVGYYPSNHIEVAS	Consensus	637–652	0.8584	Probable Non-Allergen	No	No	Yes
MTDNLQMAFVIS	Singleton	661–673	0.821	Probable Non-Allergen	No	No	Yes
IVDIQQTFFDKTWPRPIDVK	Consensus	104–124	0.8024	Probable Allergen	NA	NA	NA
GSSFGNFSANNKSGAYFN	Consensus	639–657	0.7847	Probable Allergen	NA	NA	NA
LLSNSTGTDFKD	Singleton	388–400	0.7804	Probable Non-Allergen	No	No	Yes
KNSQSSPIIPGF	Singleton	398–410	0.7804	Probable Non-Allergen	No	No	No
LALQEVVKALNESYIDLKELGNY	Consensus	502–524	0.7702	Probable Non-Allergen	No	No	No
FSDIKTHKSQPLNAG	Consensus	601–615	0.7665	Probable Non-Allergen	No	No	No
GFTTTNAFKVQ	Consensus	362–373	0.7624	Probable Allergen	NA	NA	NA
IIPGFGGNFNLKLLEPVS	Consensus	302–320	0.7494	Probable Non-Allergen	No	No	Yes
FNITEDEAEWFGITQNAQGVHL	Consensus	111–133	0.7405	Probable Non-Allergen	No	No	Yes
EHDEWFGITQDT	Singleton	399–410	0.7036	Probable Allergen	NA	NA	NA
FQNISNLPPPLLN	Consensus	1134–1147	0.7032	Probable Non-Allergen	No	No	Yes
IDCGHDDLAQLRCS	Consensus	456–471	0.6975	Probable Allergen	NA	NA	NA
EQAEGVECDFSLLL	Consensus	815–829	0.6827	Probable Non-Allergen	No	No	Yes
DQLNSTYFKLSI	Singleton	771–783	0.6751	Probable Non-Allergen	No	No	Yes
DGIVIRIGQNANKTGSVI	Consensus	823–841	0.6684	Probable Allergen	NA	NA	NA
LAFSKVQEAVNA	Singleton	697–709	0.668	Probable Allergen	No	No	No
LPPPLLNNQTDL	Singleton	1275–1287	0.658	Probable Non-Allergen	No	No	Yes
MAATGVISSMTDNLQMAF	Consensus	1134–1151	0.634	Probable Allergen	NA	NA	NA
EQAEGFECDFSPLLSGTPPQV	Consensus	786–807	0.6256	Probable Non-Allergen	No	No	Yes
DKSWPRPIDPAAA	Consensus	965–988	0.6222	Probable Allergen	NA	NA	NA
IIPGFGGDFNLT	Singleton	602–614	0.622	Probable Non-Allergen	No	No	Yes
FMLGSSVGNFSNGS	Consensus	236–250	0.6195	Probable Non-Allergen	No	No	Yes
GYHPSQHIEVVA	Singleton	404–416	0.6166	Probable Non-Allergen	No	No	Yes
LFASVKSSQSSPII	Consensus	345–359	0.6133	Probable Allergen	NA	NA	NA
AGYKVLPPLYDPNMEAAYTSSL	Consensus	928–950	0.6025	Probable Non-Allergen	No	No	Yes
IIPHSIRSIQSDRKAWAAFYVYKLQPLTF	Consensus	887–901	0.6015	Probable Non-Allergen	No	No	Yes
STGSRSARSAIEDLLFKVTIADP	Consensus	125–147	0.5925	Probable Non-Allergen	No	No	Yes
IGAAANSTGTVIISP	Consensus	625–639	0.5861	Probable Allergen	NA	NA	NA
FVYDFDNIG	Consensus	114–123	0.5855	Probable Non-Allergen	No	No	Yes
GQSLCALPDTPSTLTPRSVRSVPGEMRLAS	Consensus	732–760	0.5429	Probable Non-Allergen	No	No	Yes
FSAYSGDIPHYVQPGQYTP	Consensus	507–525	0.54	Probable Non-Allergen	No	No	Yes
EFSCDGISPDAI	Singleton	687–699	0.5325	Probable Allergen	NA	NA	NA
ATDCSDGNYNRNASLNSF	Consensus	514–531	0.5206	Probable Allergen	NA	NA	NA
ACEHITTMMQFS	Consensus	634–644	0.5155	Probable Allergen	NA	NA	NA
GSSFYAPEPITSLNTKYVAPQ	Consensus	333–355	0.4965	Probable Allergen	NA	NA	NA
DGYIRRAIDCGFNDLSQLCSYE	Consensus	367–386	0.4774	Probable Non-Allergen	No	No	Yes
KITIADPGYMQGYDDCMQQGPASARDLICAQYVAG	Consensus	896–931	0.4675	Probable Non-Allergen	No	No	Yes
FAYPLSMKSYMQ	Singleton	281–293	0.4593	Probable Allergen	NA	NA	NA
KNVSSQGPNFQE	Singleton	205–217	0.4589	Probable Non-Allergen	No	No	Yes
IFATAPANLTISKPSSYS	Consensus	976–994	0.4536	Probable Non-Allergen	No	No	Yes
EPIDMNKADGVIYPGRTYS	Consensus	231–248	0.4392	Probable Allergen	NA	NA	NA
LFVEDCLPLGQSLCA	Consensus	967–692	0.4392	Probable Allergen	NA	NA	NA
EMCLASIAFNHPIQVDQLNSSYFK	Consensus	850–873	0.4138	Probable Non-Allergen	No	No	Yes
HFVYDAYNLVGYYSDDGNYYCV	Consensus	233–253	0.412	Probable Allergen	NA	NA	NA
GSSVGNYYNGYP	Singleton	1041–1053	0.4007	Probable Non-Allergen	No	No	Yes
GCSVGNFSDGKM	Singleton	783–795	0.3891	NA	NA	NA	NA
LILDYFSYPLSMKSDLSVSSS	Consensus	364–384	0.388	NA	NA	NA	NA
FATYHTPATDCSDGNY	Consensus	532–549	0.3792	NA	NA	NA	NA
ITTFMPQFSRMTQSALRMR	Consensus	1041–1061	0.3727	NA	NA	NA	NA
FGAISASIGDIIQRLDLEQDAQIDRLI	Consensus	366–387	0.3635	NA	NA	NA	NA
GNHCPAGNSYTSFATYHT	Consensus	1265–1283	0.3452	NA	NA	NA	NA
CPKEFANDTKIASQLGN	Consensus	146–164	0.3352	NA	NA	NA	NA
NAKADGIIYPTGKSYSNI	Consensus	541–557	0.3262	NA	NA	NA	NA
ILPPPLLSNST	Consensus	442–452	0.3216	NA	NA	NA	NA
LLGNSXGIDFQDELDEFFKNVSTSIPNFGS	Consensus	951–981	0.3057	NA	NA	NA	NA
CVLGLVNSSLVEDCK	Consensus	712–728	0.3009	NA	NA	NA	NA
GNMFRFASLPVY	Singleton	463–475	0.2847	NA	NA	NA	NA
DQLNSSYKLSIP	Singleton	771–784	0.284	NA	NA	NA	NA
CGISPDAIARGCYS	Consensus	374–387	0.2786	NA	NA	NA	NA
LDFKEELEEFFK	Singleton	856–567	0.2785	NA	NA	NA	NA
ASAYGLCDAANPTNCIAPVNG	Consensus	784–805	0.2607	NA	NA	NA	NA
DAVNNAQALSKLAS	Consensus	459–463	0.2396	NA	NA	NA	NA
AAIASNCYSSLID	Consensus	931–943	0.2103	NA	NA	NA	NA
LLGSIAGAGWTAGLSSFAAI	Consensus	98–117	0.1785	NA	NA	NA	NA
GYFIKTNNTIVDEWS	Consensus	114–128	0.1375	NA	NA	NA	NA
AANSTGNLIISSS	Consensus	1187–1198	0.1231	NA	NA	NA	NA
FDNIIGFHSDDGNYY	Consensus	604–619	0.1103	NA	NA	NA	NA
DVSKADGIIPQ	Consensus	187–198	0.0638	NA	NA	NA	NA
AKINQALHGANLRQDSVRNL	Consensus	116–135	0.0607	NA	NA	NA	NA
KLIANKFNQALGAMQTGF	Consensus	254–271	0.0337	NA	NA	NA	NA
HSDGNYYCVRPCVS	Consensus	456–468	−0.0086	NA	NA	NA	NA
DLYGGNMFQFATPV	Consensus	410–425	−0.0297	NA	NA	NA	NA
CNGFQKCEQLLREYGQFCA	Consensus	435–448	−0.0311	NA	NA	NA	NA
ITKPLKYSYINKCSRLLSDDRTEVPQ	Consensus	491–515	−0.0554	NA	NA	NA	NA
LMQDESVANLFSDIKTHKS	Consensus	639–657	−0.0873	NA	NA	NA	NA
EKLLEQYGQFCS	Singleton	523–534	−0.0925	NA	NA	NA	NA
AFVAQQLVRSEAAR	Consensus	149–161	−0.1895	NA	NA	NA	NA
MYLYSAAHADPNRFILGKLY	Consensus	84–104	−0.3463	NA	NA	NA	NA
GFAKCEKLLEQY	Singleton	968–980	−0.3673	NA	NA	NA	NA
DQSFKDELEEFF	Singleton	1214–1240	−0.3752	NA	NA	NA	NA
EQEVQIDRLING	Singleton	987–997	−0.4307	NA	NA	NA	NA

**Table 2:** The refined models of the vaccine construct generated by the galaxyweb server. Almost five models are generated from the predicted vaccine structure, showing comparative clash and stability scores (last column, model). The top model has the lowest clash score and Root Mean Square Deviation (RMSD), and the highest global distance test–high accuracy (GDT-HA) model value was selected for further analysis.

Model	GDT-HA	RMSD	MolProbity	Clash Score	Poor	Model
Initial	1.0000	0.000	3.802	124.4	6.8	87.3
MODEL 1	0.9110	0.502	2.197	17.6	0.3	92.9
MODEL 2	0.9047	0.530	2.336	20.8	1.3	93.4
MODEL 3	0.9085	0.520	2.260	21.1	1.0	93.1
MODEL 4	0.9097	0.512	2.175	19.9	1.0	94.4
MODEL 5	0.9066	0.513	2.225	19.4	1.0	93.1

**Table 3:** Pairs of amino acid selected for disulfide bonds. # represents residue number.

Residues1 Seq #	Residues1 AA	Residues2 Seq #	Residues2 AA	Chi3	Energy
3	PRO	40	ILE	101.55	4.59
8	ASP	14	HIS	126.98	3.65
17	GLN	20	THR	91.37	0.67
40	ILE	45	ASN	119.1	6.36
89	TRP	94	PRO	113.14	4.49
94	PRO	108	ALA	−67.22	1.94
100	ILE	104	ASN	119.75	1.75
114	ASP	117	TYR	−115.58	5.25
117	TYR	119	GLN	−104.25	3.99
135	PHE	139	GLY	−88.2	3.57
140	PRO	158	ASN	−71.39	5.81
144	VAL	150	SER	77.6	3.93
146	GLN	149	SER	86.9	3.39
161	TYR	170	PRO	101.78	4.59
163	LYS	167	GLY	97.48	3.31
164	LEU	167	GLY	114.04	1.57
166	ILE	189	PRO	−78.73	6.1
168	PRO	177	ASN	108.77	2.71
171	GLY	174	ASN	−111.44	7.95
175	ILE	178	LEU	−92.5	4
183	PRO	217	LEU	−103.94	5.86
218	ASN	237	ASN	−116.83	6.08
228	GLY	232	PRO	−68.03	2.7
234	PHE	238	PHE	−63.92	6.41
247	PRO	257	PRO	121.42	3.06
250	GLY	290	GLY	−86.8	3.77
253	PHE	256	GLY	−91.29	5.01
260	GLY	263	ILE	121.84	6.03
295	ASP	321	PHE	−88.18	2.56
309	CYS	321	PHE	−77.33	1.61
310	ASP	313	LEU	107.72	5.77
313	LEU	318	GLY	108.9	2.53
322	MET	336	ARG	−61.42	5.7
324	GLY	335	GLY	−86.84	4.62
325	SER	328	GLY	102.97	4.66
339	ILE	360	TRP	−65.91	2.85
348	GLY	353	ILE	84.25	3.34
362	PRO	366	MET	80.19	6.16
378	GLY	381	ASP	−112.52	3.12

**Table 4:** The docking scores of the vaccine and Toll-like receptor 3. Different clusters of amino acids contributing to docking are shown. The top cluster has 47 amino acids involved, which have a central lowest energy of −1000.4 kcal/mol and a comparative overall lowest energy of −1033.2 kcal/mol, respectively. The lower the binding energy, the more tightly the vaccine will bind to the immune receptors.

Cluster	Members	Representative	Weighted Score
0	47	Center	−1000.4
		Lowest Energy	−1033.2
1	37	Center	−835.7
		Lowest Energy	−835.7
2	30	Center	−890.0
		Lowest Energy	−975.4
3	27	Center	−813.8
		Lowest Energy	−903.8
4	24	Center	−801.0
		Lowest Energy	−888.1
5	19	Center	−906.6
		Lowest Energy	−946.3
6	19	Center	−902.1
		Lowest Energy	−902.1
7	18	Center	−881.3
		Lowest Energy	−886.3
8	18	Center	−960.8
		Lowest Energy	−960.8
9	17	Center	−900.9
		Lowest Energy	−918.8
10	16	Center	−853.9
		Lowest Energy	−940.1
11	15	Center	−839.4
		Lowest Energy	−951.9
12	14	Center	−792.0
		Lowest Energy	−911.9
13	13	Center	−943.0
		Lowest Energy	−943.0
14	12	Center	−905.4
		Lowest Energy	−905.4
15	12	Center	−882.5
		Lowest Energy	−883.4
16	12	Center	−851.5
		Lowest Energy	−851.5
17	12	Center	−837.8
		Lowest Energy	−1040.0
18	11	Center	−1033.1
		Lowest Energy	−1033.1
19	11	Center	−881.3
		Lowest Energy	−881.3
20	11	Center	−871.7
		Lowest Energy	−871.7

**Table 5:** The docking scores of the vaccine and Toll-like receptor 9. Different clusters of amino acids contributing to docking are shown. The top cluster has 47 amino acids involved, which have a central lowest energy of −1036.7 kcal/mol and a comparative overall lowest energy of −1203.3 kcal/mol, respectively. The lower the binding energy, the more tightly the vaccine will bind to the immune receptors.

Cluster	Members	Representative	Weighted Score
0	30	Center	−1036.7
		Lowest Energy	−1203.3
1	24	Center	−1318.0
		Lowest Energy	−1318.0
2	23	Center	−1068.5
		Lowest Energy	−1228.9
3	22	Center	−995.4
		Lowest Energy	−1147.7
4	21	Center	−1068.5
		Lowest Energy	−1298.6
5	21	Center	−1055.6
		Lowest Energy	−1172.7
6	21	Center	−1009.1
		Lowest Energy	−1295.4
7	20	Center	−1212.1
		Lowest Energy	−1242.1
8	19	Center	−991.7
		Lowest Energy	−1338.5
9	18	Center	−1016.1
		Lowest Energy	−1206.8
10	18	Center	−1140.1
		Lowest Energy	−1217.3
11	16	Center	−1030.0
		Lowest Energy	−1424.7
12	16	Center	−1199.1
		Lowest Energy	−1199.1
13	16	Center	−1150.4
		Lowest Energy	−1300.8
14	16	Center	−1125.0
		Lowest Energy	−1155.1
15	14	Center	−1138.0
		Lowest Energy	−1138.0
16	14	Center	−1084.5
		Lowest Energy	−1122.5
17	13	Center	−1108.6
		Lowest Energy	−1151.3
18	13	Center	−1215.2
		Lowest Energy	−1215.2
19	13	Center	−1047.6
		Lowest Energy	−1095.5
20	13	Center	−1003.2
		Lowest Energy	−1218.4

## Data Availability

The data generated in the work is presented in the manuscript.
